# Collecting Food and Drink Intake Data With Voice Input: Development, Usability, and Acceptability Study

**DOI:** 10.2196/41117

**Published:** 2023-03-31

**Authors:** Louise A C Millard, Laura Johnson, Samuel R Neaves, Peter A Flach, Kate Tilling, Deborah A Lawlor

**Affiliations:** 1 Medical Research Council (MRC) Integrative Epidemiology Unit University of Bristol Bristol United Kingdom; 2 Department of Population Health Sciences Bristol Medical School University of Bristol Bristol United Kingdom; 3 Centre for Exercise, Nutrition and Health Sciences School for Policy Studies University of Bristol Bristol United Kingdom; 4 Centre for Health National Centre for Social Research (NatCen) London United Kingdom; 5 Faculty of Engineering University of Bristol Bristol United Kingdom

**Keywords:** digital health, data collection, voice-based approaches, Amazon Alexa, self-reported data, food and drink

## Abstract

**Background:**

Voice-based systems such as Amazon Alexa may be useful for collecting self-reported information in real time from participants of epidemiology studies using verbal input. In epidemiological research studies, self-reported data tend to be collected using short, infrequent questionnaires, in which the items require participants to select from predefined options, which may lead to errors in the information collected and lack of coverage. Voice-based systems give the potential to collect self-reported information “continuously” over several days or weeks. At present, to the best of our knowledge, voice-based systems have not been used or evaluated for collecting epidemiological data.

**Objective:**

We aimed to demonstrate the technical feasibility of using Alexa to collect information from participants, investigate participant acceptability, and provide an initial evaluation of the validity of the collected data. We used food and drink information as an exemplar.

**Methods:**

We recruited 45 staff members and students at the University of Bristol (United Kingdom). Participants were asked to tell Alexa what they ate or drank for 7 days and to also submit this information using a web-based form. Questionnaires asked for basic demographic information, about their experience during the study, and the acceptability of using Alexa.

**Results:**

Of the 37 participants with valid data, most (n=30, 81%) were aged 20 to 39 years and 23 (62%) were female. Across 29 participants with Alexa and web entries corresponding to the same intake event, 60.1% (357/588) of Alexa entries contained the same food and drink information as the corresponding web entry. Most participants reported that Alexa interjected, and this was worse when entering the food and drink information (17/35, 49% of participants said this happened often; 1/35, 3% said this happened always) than when entering the event date and time (6/35, 17% of participants said this happened often; 1/35, 3% said this happened always). Most (28/35, 80%) said they would be happy to use a voice-controlled system for future research.

**Conclusions:**

Although there were some issues interacting with the Alexa skill, largely because of its conversational nature and because Alexa interjected if there was a pause in speech, participants were mostly willing to participate in future research studies using Alexa. More studies are needed, especially to trial less conversational interfaces.

## Introduction

Epidemiological cohorts typically collect data at widely spaced time points (eg, every 1-5 years) [1,2]. Although some types of traits (eg, weight or height) are fairly stable or change gradually over time, others such as activity levels, blood glucose levels, mental well-being, and dietary intake can vary more acutely, for example, within days, hours, or even minutes. For these traits, prospectively capturing how they vary across time allows us to assess how this variability relates to other traits and disease. Some acutely varying traits can be collected continuously and objectively using wearable digital devices; for example, physical activity can be tracked using accelerometers or blood glucose can be measured using continuous glucose monitors [3]. For others, such as mental health traits and dietary intake, no objective approach to measuring within-day variation in these traits exists, and they need to be collected by self-report.

One possible approach to providing real-time self-reported information is verbal input, which could enable participants to conveniently enter free text. Over the last few years, several technology companies have released voice-controlled “smart” systems. These systems, such as Amazon Alexa, Google Assistant, and Samsung’s Bixby, allow users to talk to a device rather than typing or pressing a button. They each have core functionality available by default (eg, saying the time when asked) and have developer platforms that allow anyone to produce and publish a custom voice-based app. This means that it is now technically possible to collect self-reported data continuously over a day or several days using verbal input.

Voice-based data collection may be most useful for collecting self-reported data that are both complex and variable across a day. One possible example is the food and drink consumed by a person and the time when they consume it. Traditionally, cohorts have collected dietary intake information using paper or web-based food frequency questionnaires or (less commonly) diaries. The limitations of these include retrospective recording, requiring conversion to an electronic form, potential for missing data because participants are not prompted for missing information, and the inconvenience of having to carry a diary. More recently, other approaches have been developed such as web-based dietary recall tools [4] and approaches using photographs [5-7]. Although these methods can collect detailed dietary information, they are burdensome, so they can only be used for short periods by highly motivated participants [3]. Approaches have been developed to detect eating events using wearable devices [8,9], for example, using wrist-worn accelerometers and gyroscopes [8]—these detect when an event occurs and not what was consumed. Wearable camera devices that capture images throughout the day have been trialed, but identifying and classifying food in images is challenging [10].

In this pilot study, we explored the potential of voice-based data collection in epidemiological research using food and drink diaries as an exemplar. Epidemiology studies are a challenging potential application of voice-based data collection because they are used to inform health policy and medical interventions; therefore, it is important to understand the biases in the collected data (eg, which food and drinks can be recorded correctly vs with error) to avoid incorrect conclusions being made. In addition, participation in epidemiological studies is predominantly altruistic, with participants usually receiving little direct benefit from participation, such that these studies aim to minimize participant burden to maximize participation. Our study has three key aims: (1) to demonstrate the technical feasibility of collecting data using Alexa, (2) to gain initial insight into participant acceptability, and (3) to provide an initial evaluation of the validity of the collected data. In general, we view the capture and processing of information as separate steps and, in this study, focused on demonstrating and evaluating the former.

## Methods

### Ethics Approval

Ethics approval was obtained from the University of Bristol Faculty of Health Sciences Research Ethics Committee (approval number 63861).

### Study Participants

Power calculations based on 2 measures suggested that a sample size of at least 35 is needed (see details in Section S1 in Multimedia Appendix 1). We recruited volunteers from the University of Bristol staff and student email lists. Participants were compensated with a £30 (US $36) voucher after they submitted the postparticipation questionnaire (receiving the voucher was not dependent on them submitting any food diary entries).

### Description of the System Architecture: a Voice-Based System Using Amazon Alexa

**Figure 1:**
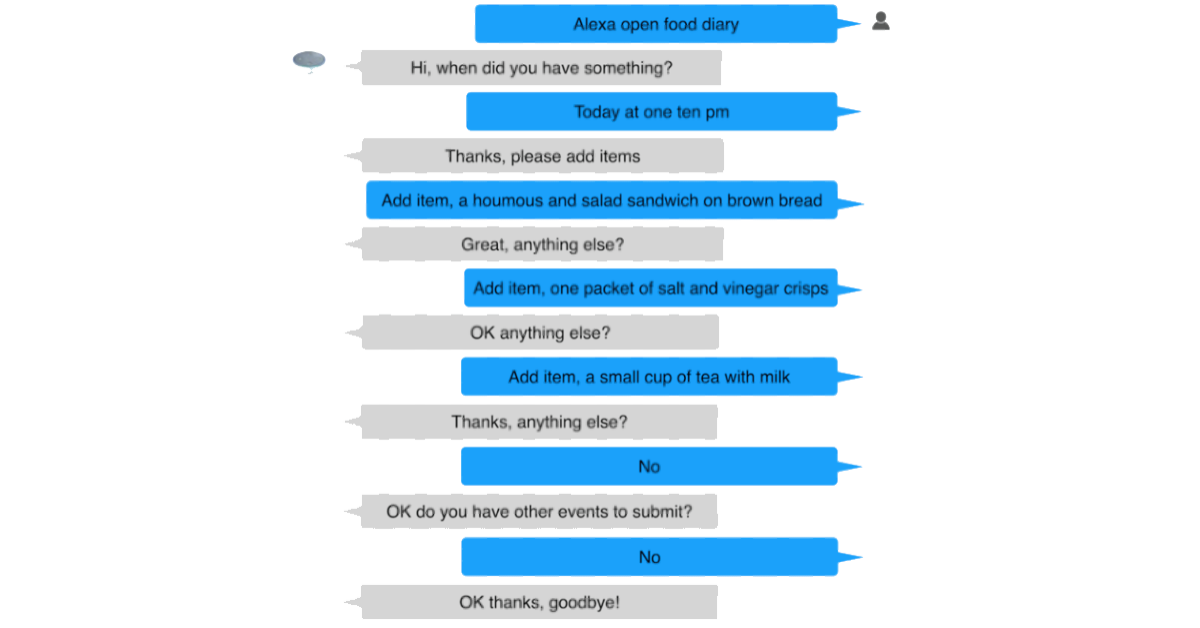
Example conversation of study participant with custom food diary skill.

In this study, we used the Amazon Alexa voice system (a comparison with other voice-based systems such as Google Assistant and Samsung’s Bixby is left for future work). The Alexa system enables the development of custom functionality, referred to as a custom skill. Alexa skills comprise intents that each define an interaction that a user can have with the skill. We developed a custom skill to collect food and drink intake events, with intents that allow participants to (1) add the date and time of an intake event, (2) add ≥1 items they ate or drank at this time, (3) cancel the event, (4) cancel the last item added to the event, and (5) submit the event. See example utterances in Table S1 in Multimedia Appendix 1 and an example conversation in Figure 1. Section S2 in Multimedia Appendix 1 provides further details on the system architecture.

### Data Collection Protocol

**Figure 2:**
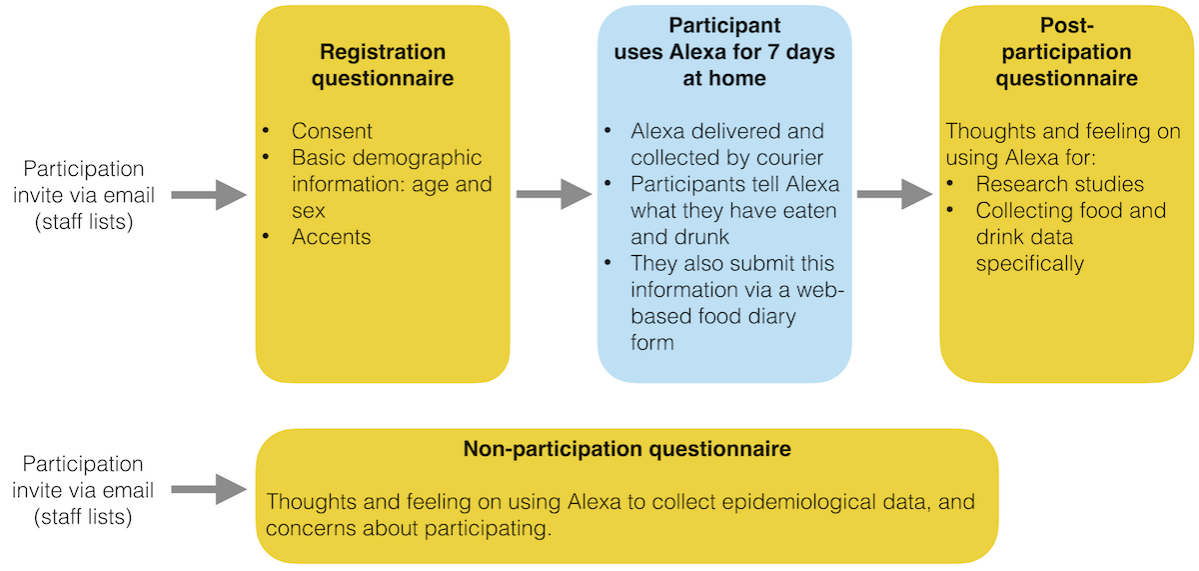
Overview of data collection protocol.

An overview of the data collection protocol is shown in Figure 2. Owing to the COVID-19 pandemic, participants took part at home. We sent an initial email with an accompanying participation information sheet (Multimedia Appendix 2) inviting staff and students to participate in this study. Upon replying, participants were sent a preparticipation questionnaire asking for basic demographic information such as their age and sex (questionnaire 1 in Multimedia Appendix 3). On completion, participants were booked for a 7-day data collection period using Alexa.

The equipment was stored in the principal investigator’s (LACM) home. On day 1 of the participants’ data collection period using Alexa, the equipment was delivered to their home by courier, along with a participant guide (Multimedia Appendix 4). The participant was asked to set up the equipment and start using it as soon as possible. Participants were instructed with the following statement: “After you have had something to eat or drink, we would like you to submit your food and drink information to Alexa first, and then submit it on the web form.” Entering the food and drink information using both Alexa and a web form (questionnaire 4 in Multimedia Appendix 3) allowed us to compare the data entered using these approaches (ie, relative validity [11]). As participants entered the date and time of the intake event, they were able to enter events consumed earlier on the same day or on a previous day (including those consumed outside the home). On day 7, the equipment was returned to the principal investigator’s home via courier. Participants were then asked to complete a postparticipation questionnaire on their experiences during the study and the acceptability of using Alexa (questionnaire 2 in Multimedia Appendix 3).

To understand views on the acceptability of using voice-based interfaces more widely (beyond our participant group), we also sent a further invitation (to the same email lists) asking those who did not participate to complete a short questionnaire about their feelings on using voice-based devices and their reasons for not participating (questionnaire 3 in Multimedia Appendix 3).

The questionnaires were deployed via the University of Bristol REDCap (Research Electronic Data Capture; Vanderbilt University) secure web platform [12]. The content of the study emails is provided in Multimedia Appendix 5.

### Analytical Sample

A participant flow diagram is shown in Figure S1 in Multimedia Appendix 1. Of the 45 participants who registered to participate, 1 (2%) withdrew and 7 (16%) were excluded owing to equipment issues (Section S3 in Multimedia Appendix 1). The remaining 82% (37/45) of participants comprised our analytical sample. Among these, 3% (1/37) of participants did not attempt to use Alexa. In addition, 19% (7/37) of participants had Alexa entries but no web diary entries completed within the 30 minutes directly following the Alexa submission. As Alexa and web entries must be submitted within 30 minutes to be identified as corresponding to the same intake event in our data processing approach (see below), the Alexa and web entries from these participants could not be compared. The entries from the remaining 78% (29/37) of participants were used to compare the information entered via the web form versus Alexa (“comparison” sample).

### Data Preprocessing

#### Mapping Web Food Form Entries to Alexa Intake Events

The web and Alexa entries both included the following information: (1) intake timestamp—the date and time the participant (said they) ate or drank; (2) submission timestamp—the date and time the participant submitted the entry; and (3) intake items—≥1 food and drink intake items. To compare the content of the web and Alexa entries, we first undertook an automated process to identify Alexa and web entry pairs that correspond to the same intake event, referred to as counterpart entries. This was nontrivial because a participant might not have entered each entry with the web form immediately after entering it via Alexa or the intake timestamp entered via Alexa might have been recorded incorrectly (ie, Alexa might have heard the day or time stated by the participant incorrectly).

We identified counterpart entries using intake and submission timestamps. The process we used was as follows (illustrated in Figure S5 in Multimedia Appendix 1):

1. Identify counterparts as the set of entries in which the web and Alexa intake timestamps were within 5 minutes of each other, and the Alexa submission timestamp was up to 30 minutes before the web submission timestamp. The nonexact match of the intake time was because participants can tell Alexa this using a phrase such as “just now” or “ten minutes ago,” which may not correspond exactly to the intake time entered using the web form.
2. Identify web counterpart entries of the Alexa submissions not matched in step 1 as the nearest subsequent web entry where one occurs within 30 minutes of the Alexa entry.

#### Comparing Food and Drink Descriptions in counterpart Web and Alexa Entries

We compared counterpart entries using 2 approaches, an automated approach and a systematic manual approach.

##### Automated Approach

We compared the text content of the counterpart entries by comparing the set of words contained in each. Entries were preprocessed to remove plurality of words (eg, “crisps” becomes “crisp”) [13] and convert numbers to numeric values (eg, “one” and “a” both become 1). For each counterpart pair, we calculated the number of words in (1) the web word set but not the Alexa word set, (2) the Alexa word set but not the web word set, and (3) both word sets.

##### Systematic Manual Approach

Our systematic manual approach was conducted by LACM. As this approach has some degree of judgment, we also asked 5 researchers independent to the project (within the same unit but not involved in this study) to review 10 random entries (none repeated across researchers) so that we can evaluate the interresearcher variability of these manual evaluations.

We used a 2-step process to conduct this manual review. First, the intake items of each counterpart pair were compared to determine whether there was any similarity. If the set of items was completely different, then they were marked as most likely corresponding to different intake events (ie, the counterpart pairing did not work in this case, eg, “a cup of coffee with milk” vs “spaghetti bolognaise”). All other entries were performed in step 2.

Step 2 involved reviewing each counterpart entry and, for each, recording the number of food or drink items in a counterpart pair in the following categories:

1. Same item semantically (the 2 entries are equivalent with no additional or different information in each)
2. Same item but with different details (eg, “cup of tea” vs “mug of tea”)
3. Same item, Alexa information has less detail (eg, “cheese and salad sandwich” vs “a sandwich”)
4. Same item, Alexa item has more detail
5. Same item, misspelling in Alexa input, but still understandable, that is, there is no loss of information (eg, “to bagels” vs “two bagels”)
6. Same item, misspelling in web form input, but still understandable
7. Same item, with Alexa entry issue, in which the consumed item is still identifiable (eg, “ball of yoghurt” rather than “bowl of yoghurt”)
8. Item with major entry issue, such that it contains no food or drink information, or the main essence of the food or drink is missing (eg, a “cough with milk” rather than “coffee with milk”)
9. Extra Alexa item with major entry issue (which can happen if a participant makes a mistake or stops talking, then tries again so there is an extra item, eg, “two”)
10. Extra Alexa item that is recognizable as a food or drink (ie, should not be assigned to category 9)
11. Extra web item

Table S2 in Multimedia Appendix 1 shows some example assignments using this approach.

The independent researchers who completed 10 entries were provided with an information sheet describing the task (Multimedia Appendix 6). We visually evaluated the agreement between the assignments of LACM and independent researchers using a stacked bar chart.

The automated and systematic manual approaches are complementary because the former is objective but is likely to be a more pessimistic assessment of agreement. This is because participants may not write an entry in the same way that they would speak it. For example, a participant might write “1 x apple. 1 bar of chocolate” but say “one apple and a chocolate bar,” which has differences in the words used even though they are semantically the same.

### Statistical Analyses

#### Use Summary

We summarized the participants’ use of the web and Alexa approaches using the median and IQR of the number of submitted web and Alexa entries, respectively.

#### Comparison of Counterpart Diary Entries

We compared the intake timestamps in the counterpart pairs using a plot similar to a Bland-Altman plot but in which the x-axis is the intake time entered using the web form rather than the average. Assuming that the intake timestamp entered on the web form will be largely correct, this is to help show whether the intake time submitted via Alexa may be less accurate for particular times of the day. We summarized automated and systematic manual comparisons using stacked bar charts.

#### Summarizing the Number of Incomplete Attempts

The Alexa skill saves partial entries (ie, those that have not been submitted, perhaps because the internet connection was interrupted) in addition to completed entries. We estimated the median (IQR) number of unsuccessful attempts across participants.

#### Evaluating Participant Questionnaire Responses on Usability and Acceptability

We summarized the responses to the postparticipation questionnaire (questionnaire 2 in Multimedia Appendix 3) and the nonparticipation questionnaire (questionnaire 3 in Multimedia Appendix 3) by calculating the number of participants (and percentage) that responded to each questionnaire item option. Responses to free-text items were read and reread to identify the key themes.

The Alexa skill and web service code, and analysis code, are publicly available [14,15]. Git tag version 0.1 of the analysis code corresponds to the version of the analyses presented here.

## Results

### Participant Characteristics

The participant characteristics are summarized in Table 1. Most participants (30/37, 81%) were in their early adulthood (aged 20-39 years). Our sample included more female participants than male participants (23/37, 62% female). The majority (31/37, 84%) reported that they did not believe they had a strong regional UK accent, with 68% (25/37) reporting that they did not have an accent because English was a second language. In total, 43% (16/37) of participants have an Alexa device at home that they use.

**Table 1:** Participant demographics (n=37).

Participant characteristics^a^		Values, n (%)
**Age range (years)**		
	<20	4 (11)
	20-29	16 (43)
	30-39	14 (38)
	40-49	2 (5)
	50-59	1 (3)
**Sex**		
	Female	23 (62)
	Male	14 (38)
	Other	0 (0)
**Has a regional UK accent**		
	No	31 (84)
	A little	4 (11)
	Yes	2 (5)
**Has a non-English accent**		
	No	25 (68)
	A little	9 (24)
	Yes	3 (8)
**Has a voice-controlled device**		
	No	16 (43)
	Yes, but it is used by others, not me	5 (14)
	Yes, and I use it	16 (43)

^a^The characteristics shown are those collected in this study.

### Use Summary

On average, participants completed more web diary entries than Alexa entries (median number of entries was 17, IQR 13-27 compared with 11, IQR 7-21; paired 2-tailed *t* test *P* value <.001; comparison shown in Figure S6 in Multimedia Appendix 1). The median number of partial Alexa attempts across all participants was 6 (IQR 1-9).

### Comparison of Counterpart Diary Entries

#### Intake Timestamp Comparison of Web Form Versus Alexa Entries

Across the 29 participants in the comparison subsample, there were 310 counterpart entries. Of these, 71.6% (222/310) had a matching timestamp (Figure S7 in Multimedia Appendix 1). The median proportion of completed counterpart entries with a matching timestamp across the participants was 0.67 (IQR 0.5-1).

#### Food and Drink Description Comparison of Web Form Versus Alexa Entries

**Figure 3:**
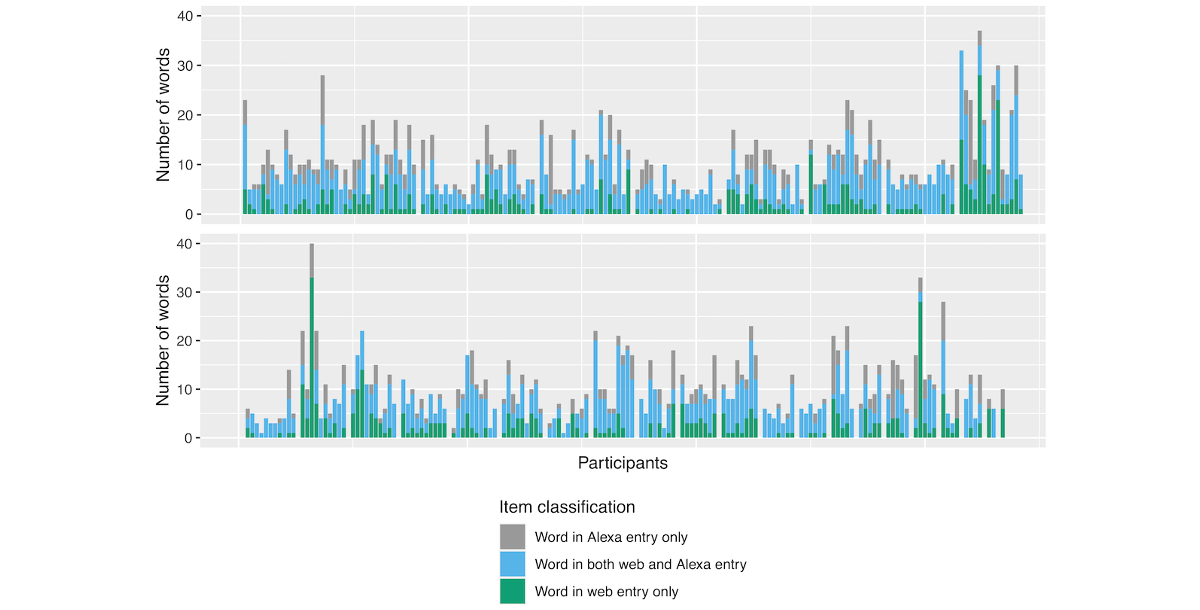
Summary of automated comparison of the food and drink information submitted using Alexa versus the web form. Results shown for 29 participants in the “comparison” sample. Each stacked bar shows the number of unique words in (1) the Alexa entry only, (2) both the web and Alexa entries, and (3) the web entry only. Each block of stacked bars shows the set of entries for a given participant.

**Figure 4:**
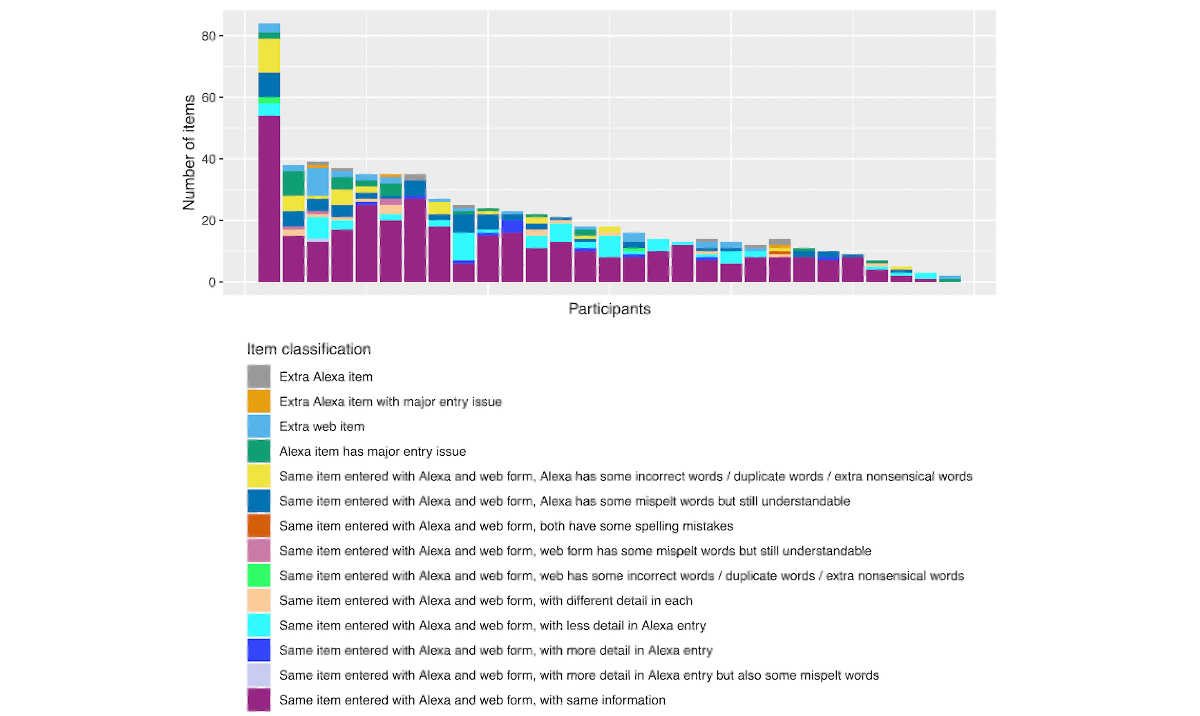
Summary of manual evaluation of the submitted food and drink information. Results shown for 29 participants in the “comparison” sample. Each stacked bar indicates the number of items in each category for each participant. Manual evaluation was conducted by LACM.

The results comparing the submitted food and drink information using automated and manual comparison approaches are shown in Figures 3 and 4, respectively. Of the 310 counterpart entries manually reviewed, 21 (6.8%) were classified as corresponding to different intake events. The remaining 93.2% (289/310) of counterpart entries included 612 web form items and 588 Alexa items, with 33 extra web items (not identified in the counterpart Alexa entry) compared with 9 extra Alexa items (not found in the counterpart web entry). The majority (357/612, 58.3% and 357/588, 60.7% for the web and Alexa items, respectively) of the items entered via the web form and Alexa were the same, containing the same information. Of the 194 items that were identified as corresponding to the same intake item but containing different information, 64 (33%) had less detail from Alexa, 12 (6.2%) had more detail from Alexa, 15 (7.7%) had different detail, 3 (1.5%) had a web entry issue, 36 (18.6%) had an Alexa entry issue, 4 (2.1%) had spelling mistakes in the web version not the Alexa version, 59 (30.4%) had a misspelling in Alexa only, and 1 (0.5%) had a misspelling in both the Alexa and web input. Of the 59 items with an Alexa misspelling, 40 (68%) were owing to Alexa recording the word “to” rather than “two.” Of the 588 items entered via Alexa, 28 (5%) were classified as having a major entry issue.

We did not identify systematic differences in the assignments by LACM for the systematic manual approach compared with those of independent researchers (Figure S8 in Multimedia Appendix 1).

### Evaluating Participant Questionnaire Responses on Usability and Acceptability

The summaries of the postparticipation questionnaire responses are provided in Table 2. Of the 35 participants who completed the postparticipation questionnaire, 26 (74%) said they would be happy to use a voice-controlled system at home for future research and 28 (80%) said they would be happy to use one on a wearable device (eg, a smart watch). Alexa sometimes interjected when participants were telling her *when* they ate or drank, with 20% (7/35) of participants saying that this happened often or always and 31% (11/35) saying that this happened occasionally. Alexa often interjected when participants were telling her *what* they ate or drank, with 51% (18/35) of participants saying that this happened often or always and 34% (12/35) saying that this happened occasionally. In terms of convenience, enjoyment, and efficiency, 51% (18/35), 60% (21/35), and 43% (15/35) of participants, respectively, said that they found using Alexa “OK” or better.

Of the 35 participants who completed the postparticipation questionnaire, 25 (71%) had previously used another approach to record their food and drink intake (Table S3 in Multimedia Appendix 1). Of the 13 participants who have previously used a traditional diary (on paper or a computer), 7 (54%) found using Alexa at least as convenient, 5 (56%) found using Alexa at least as enjoyable, and 2 (22%) found using Alexa at least as efficient. Of the 34% (12/35) of participants who have previously used MyFitnessPal, 75% (9/12) found Alexa at least as convenient, 64% (7/12) found Alexa at least as enjoyable, and 36% (4/12) found Alexa at least as efficient.

**Table 2:** Postparticipation questionnaire summary (n=35).

Questionnaire items^a^		Values, n (%)
**Participant was able to accurately tell Alexa what they ate and drank (n=35)**		
	Completely agree	3 (9)
	Somewhat agree	16 (46)
	Neither agree not disagree	2 (6)
	Somewhat disagree	12 (34)
	Completely disagree	2 (6)
**Participant was able to estimate accurate quantities describing how much they ate (n=34)**		
	Completely agree	3 (9)
	Somewhat agree	11 (32)
	Neither agree not disagree	10 (29)
	Somewhat disagree	9 (26)
	Completely disagree	1 (3)
**Participant chose not to record particular snacks or meals (eg, because it was unhealthy; n=35)**		
	Completely agree	1 (3)
	Somewhat agree	5 (14)
	Neither agree not disagree	0 (0)
	Somewhat disagree	7 (20)
	Completely disagree	22 (63)
**Participant sometimes chose to be selective with the truth (n=35)**		
	Completely agree	3 (9)
	Somewhat agree	5 (14)
	Neither agree not disagree	3 (9)
	Somewhat disagree	6 (17)
	Completely disagree	18 (51)
**Participant felt they remembered to submit food and drink information (n=35)**		
	Completely agree	9 (26)
	Somewhat agree	19 (54)
	Neither agree not disagree	1 (3)
	Somewhat disagree	6 (17)
	Completely disagree	0 (0)
**Alexa interjected when I had not finished telling her when I ate or drank (n=35)**		
	Never	4 (11)
	Rarely	13 (37)
	Occasionally	11 (31)
	Often	6 (17)
	Always	1 (3)
**Alexa interjected when I had not finished telling her what I ate or drank (n=35)**		
	Never	2 (6)
	Rarely	3 (9)
	Occasionally	12 (34)
	Often	17 (49)
	Always	1 (3)
**How convenient or inconvenient did you find providing information using Alexa? (n=35)**		
	Very inconvenient	4 (11)
	Somewhat inconvenient	13 (37)
	OK	10 (29)
	Somewhat convenient	7 (20)
	Very convenient	1 (3)
**How enjoyable or unenjoyable did you find providing information using Alexa? (n=35)**		
	Very unenjoyable	3 (9)
	Somewhat unenjoyable	11 (31)
	OK	15 (43)
	Somewhat enjoyable	6 (17)
	Very enjoyable	0 (0)
**How efficient or inefficient did you find providing information using Alexa? (n=35)**		
	Very inefficient	5 (14)
	Somewhat inefficient	15 (43)
	OK	6 (17)
	Somewhat efficient	8 (23)
	Very efficient	1 (3)
**How easy or hard did you find providing information using Alexa? (n=35)**		
	Could not use at all	0 (0)
	Very hard	3 (9)
	Somewhat hard	18 (51)
	OK	7 (10)
	Somewhat easy	7 (20)
	Very easy	0 (0)
**Happy to use a voice-controlled system (eg, Alexa) at home for research in the future (n=35)**		
	Yes	26 (74)
	No	2 (6)
	Not sure	7 (20)
**Happy to use a voice-controlled system (eg, Alexa) on a wearable device such as a smart watch, for research (n=35)**		
	Yes	28 (80)
	No	1 (3)
	Not sure	6 (17)

^a^A summary of all items in the postparticipation questionnaire is provided in Table S3 in Multimedia Appendix 1.

### Evaluating Nonparticipation Questionnaire Responses

Of the 69 participants who responded, 11 (16%) did not take part because of privacy concerns (with respect to Amazon, researchers collecting their diet information, or Alexa inadvertently listening to other conversations; Table 3). In total, 61% (42/69) stated that they would be happy to use Alexa at home for future research, whereas 57% (39/69) said that they would be happy to use Alexa on a wearable device for research purposes.

**Table 3:** Nonparticipation questionnaire summary (n=69).

Questionnaire items		Value, n (%)
**Age range (years)**		
	<20	13 (19)
	20-29	36 (52)
	30-39	11 (16)
	40-49	5 (7)
	50-59	2 (3)
	Prefer not to answer	2 (3)
**Sex**		
	Female	51 (74)
	Male	18 (26)
	Other	0 (0)
**Reasons did not take part**		
	Not available during the study session times	30 (43)
	Data privacy concerns around Amazon collecting information on my diet	9 (13)
	Data privacy concerns around researchers collecting information on my diet	3 (4)
	Concerns that Alexa will inadvertently listen to other conversations	9 (13)
	I do not eat or drink during my working hours	3 (4)
	Picking up and returning the device was inconvenient	5 (7)
	Other reason^a^	21 (30)
**Has a voice-controlled device**		
	No	35 (51)
	Yes, but it is used by others, not me	10 (14)
	Yes, and I use it	24 (35)
**Happy to use a voice-controlled system (eg, Alexa) at home for research in the future**		
	Yes	42 (61)
	No	13 (19)
	Not sure	14 (20)
**Happy to use a voice-controlled system (eg, Alexa) on a wearable device such as a smart watch for research**		
	Yes	39 (57)
	No	18 (26)
	Not sure	12 (17)

^a^Participants who stated “other” were able to complete a free-text response; these are summarized in Section S4 in Multimedia Appendix 1. A summary of all items in the nonparticipation questionnaire is provided in Table S4 in Multimedia Appendix 1.

## Discussion

In this study, we have demonstrated the technical feasibility of collecting data using Alexa for epidemiological research by successfully developing an Alexa skill to collect food and drink information and using it to collect data from 37 participants across a period of 7 days (5 full days). Our results provide useful initial insight into the participant acceptability of using this approach and validity of the collected data. On average, more entries were submitted via the web form than via Alexa. Our results suggest that intake date and time was largely entered accurately via Alexa. The majority of the Alexa entries (357/588, 60.7%) contained the same food and drink information as the corresponding web entry, according to our systematic manual approach. The most common differences were Alexa information having less detail or a homophone error (most often “to” rather than “two”).

Overall, the usability of our Alexa skill was fairly poor. Most participants reported that Alexa interjected while they were trying to enter food and drink information (12/35, 34% of participants sometimes and 18/35, 51% often or always), with better results for the date or time of the intake event (11/35, 31% of participants sometimes and 7/35, 20% often or always). Several participants reported finding it difficult to avoid pausing while articulating what they ate or drank, which might cause Alexa to interject or cut out. Some reported reducing the information they provided, so that Alexa would be more likely to accept it. The participants also reported that Alexa sometimes did not understand or would exit the skill during use.

The voice interface we have trialed comprises our Alexa skill implementation and the Amazon back-end logic, and only the former is under our control. The implementation and deployment of the Alexa skill has several components, with many choices regarding the design of the voice interface, the technical infrastructure, and the study protocol (eg, location of data collection, which was home based in our study). Each of these factors may have affected the usability of the skill to collect food and drink information. Most notably, we conclude that the conversational interface of our skill (in which participants first tell Alexa the time, then each of the items consumed) was not successful, because when the skill inadvertently cut out (eg, because of multiple failed attempts to converse with Alexa or a poor internet connection), the participant would have to start that entry from the beginning. A less conversational interface in which the participant states the information without separate prompts would likely be more usable. Although our results suggest that Alexa may be more appropriate for entering short summaries of information, in the longer term, the integration of this approach with other approaches (such as a phone app) can be used to supplement voice-collected data. For example, using Alexa to log events directly after eating or drinking (eg, on a wearable device) and then entering more detail via a phone app when convenient. Therefore, while in this study, participants entered more events and provided more detail using the web form, there are some opportunities to improve the skill for future studies.

The strengths of our study include the collection of pilot data “in the wild” rather than in a controlled laboratory-based setting. We collected food and drink information via a web form, in addition to Alexa, to allow the comparison of the data collected using these approaches. Our study had several limitations. While asking participants to provide information via both Alexa and a web form was valuable, interactions with one of these approaches may have affected their interaction and perceived feelings toward the other. The Alexa and web entries in our data had no explicit link and identifying entries that corresponded to the same intake event was difficult. We could only assess the relative validity of the Alexa entries (relative to the web form entries), that is, we have no absolute ground truth. Although the intake date and time could be easily compared between the Alexa and web form entries in an automated manner, comparing the free-text food and drink information was nontrivial as differences in the way the participant conveyed this information would not necessarily amount to meaningful differences in the submitted information. Most of these limitations could be rectified by integrating this voice-based approach with a phone app in which the participant can review each entry and either correct it or mark it as correct, instead of requiring a web diary, so that validity can be assessed in an automated manner by evaluating the corrections made by the participant. This would likely increase the number of Alexa entries that could be evaluated and could also reduce the participant burden because entries would either need to be marked as correct or corrected, rather than inputting all the information on a web form. Additional strengths and limitations and details are provided in Section S5 in Multimedia Appendix 1.

Although other studies have used voice-based approaches in other health settings [16-19], to the best of our knowledge, this is the first study to assess collecting self-reported epidemiology data with a voice-based system (to the best of our knowledge, a previous grant that sought to create a voice-based interface did not achieve this objective [20]). Furthermore, although our focus was on using this technology for collecting epidemiological data, the results of our study are likely to be useful more broadly, for example, to inform the development of technologies for personalized health care or commercial systems (collecting self-reported data to track behavior).

Table 4 summarizes the main findings of this study. More studies are needed to understand the strengths and limitations of different approaches to collect epidemiological data using voice, for example, with different voice-based systems (eg, comparing Amazon Alexa vs Google Assistant), different types of devices (eg, wearables vs smartphones), different voice interface designs, particularly those that are less conversational, and to further evaluate biases in the collected data [21]. Although this study used an Amazon Echo Dot device situated in the participants’ home, it is also possible to deploy an Alexa skill on other devices, for example, on smartphones and wearables. The acceptability of collecting epidemiological data with voice (including the length of time a participant may be willing to use such an approach), and the accuracy of the collected data, may differ depending on the device used (eg, because of differing levels of background noise when “on the go” vs in the home environment). Further studies are needed to investigate this. Voice-based approaches may be particularly useful in populations that might not be able to write (or write with ease), for example, those with learning difficulties, such as dyslexia, or certain diseases, such as motor neuron disease.

**Table 4:** Summary of the main results and implications for future research.

Results	Implications for future research
Voice-based data collection is technically feasible.	Future studies are needed to understand the strengths and limitations of different voice interfaces.
Conversational interface was a frustration for users because it could cut out (eg, owing to a poor internet connection) and the conversation would have to start from the beginning.	Design a less conversational voice interface.
Alexa more suited to entering short bits of information.	Integration with a phone app would allow supplementing information to be entered with voice entries.
The majority of the Alexa entries (357/588, 60.7%) contained the same food or drink information as the corresponding web entry, but a substantial proportion contained differences.	Trial and compare different voice-based systems such as the Google Assistant.
Matching voice entry with corresponding web form entry was difficult and many could not be matched (and therefore compared).	Use a phone app to evaluate the collected data by asking participant to validate the entry, either marking the entry as correct or providing a correction.

## Appendix

Multimedia Appendix 1 Supplementary text, figures, and tables.

Multimedia Appendix 2 Study participant information sheet.

Multimedia Appendix 3 Study questionnaires.

Multimedia Appendix 4 Study participant guide.

Multimedia Appendix 5 Study emails.

Multimedia Appendix 6 Information sheet describing manual comparison task, of Alexa, and web entries.

## Abbreviations

REDCap: Research Electronic Data Capture
