# Supplementary material for: Collecting Food and Drink Intake Data With Voice Input: Development, Usability, and Acceptability Study
Source: JMIR Mhealth Uhealth. 2023 Mar 31;11:e41117. doi: 10.2196/41117 (PMC10132002; doi:10.2196/41117)
Supplement: Multimedia Appendix 1 [file mhealth_v11i1e41117_app1.pdf]

Collecting food and drink intake data with voice input: Development, usability, and acceptability study

Millard et al.

MULTIMEDIA APPENDIX 1: SUPPLEMENTARY TEXT, FIGURES AND TABLES

TABLE OF CONTENTS

SUPPLEMENTARY TEXT 2
Supplementary section S1: Power calculations to determine appropriate sample size 2
Supplementary section S2: Technical details of system architecture 2
Supplementary section S3: Analytical sample 3
Supplementary section S4: Summaries of responses to free-text questionnaire items 3
Supplementary section S5: Further study strengths and limitations 4
SUPPLEMENTARY FIGURES 6
Supplementary figure S1: Participant flow diagram 6
Supplementary figure S2: Infrastructure of custom Alexa skill 7
Supplementary figure S3: System architecture 7
Supplementary figure S4: User journey of food diary skill 8
Supplementary figure S5: Food diary data processing approach 9
Supplementary figure S6: Summaries of the number of diary entries entered via the web form and Alexa 10
Supplementary figure S7: Plot comparing intake timestamps recorded using Alexa versus the web form 11
Supplementary figure S8: Comparison of systematic manual approach assessments of LACM versus independent assessors 12
SUPPLEMENTARY TABLES 13
Supplementary table S1: Skill intents and example utterances 13
Supplementary table S2: Examples of manual text comparison 14
Supplementary table S3: Post-participation questionnaire summary 15
Supplementary table S4: Non-participation questionnaire responses 18

SUPPLEMENTARY TEXT

Supplementary section S1: Power calculations to determine appropriate sample size

As described in the introduction we aimed to assess both technological feasibility and participant acceptability. We defined primary measures of each of these on which to base our power calculations.

Acceptability
Primary measure: the proportion of participants that said they would be happy to use Alexa for research in the future (for both home and body-worn devices).
Power calculation statistic: Precision of the primary measure
Anticipated true proportion: 0.9
Desired precision (half width of CI): 0.1 (i.e. CI: 0.80-0.90]
Confidence level: 0.95
Sample size required: 35
Calculated using: https://epitools.ausvet.com.au/oneproportion

Feasibility
Primary measure of feasibility: the average accuracy of submitted food and drink data compared to web form (i.e. the proportion of intake events containing the same information).
Power calculation statistic: precision of the primary measure.
Anticipated true average accuracy: 0.8
Desired precision (half width of CI): 0.05
Sample size required: **22**
Calculated with Stata code: ciwidth onemean, probwidth(0.8) width(0.1) sd(0.1)

Supplementary section S2: Technical details of system architecture

In this study we use the Amazon Alexa voice system as it is used most widely and has the most mature developer capabilities (in comparison with the Google Assistant).

Custom skills are set up using the Alexa Skills Kit, which comprises of the Alexa developer console, an application programming interface (API), documentation and code samples [https://developer.amazon.com/en-US/alexa/alexa-skills-kit]. The general infrastructure required for an Alexa skill is shown in Supplementary figure S2, where a custom skill is installed on an Alexa enabled device. When the skill is used, the audio is sent to the Alexa service in the cloud for processing. The Alexa skill, set up by the developer in the Alexa Developer Console, processes the audio input and then textual data is sent to a backend web service, also set up by the developer, that hosts the backend conversational logic of the Alexa skill. We use a Lambda function on the Amazon Web Service (AWS) to host the web service and use a MariaDB database hosted on the Relational Database Service of the AWS to store the collected data. An overview of the system design is shown in Supplementary figure S3.

System configuration to improve response speeds
Warm starts: A skill using a lambda function can be slow to start if the lambda function needs to be reprovisioned before it is ready. We use a CloudWatch timer to keep the lambda function “warm” so that response times are minimised.

Memory and CPU provisioning: We increased the lambda memory from 512MB to 1024MB which also increases the CPU power “proportionately”.

Supplementary section S3: Analytical sample

Of the 7 participants excluded with incorrect equipment, 4 were due to an equipment delivery error where the wrong equipment boxes were delivered to the wrong participants such that their web food diaries were mixed up and could not be used. The other 3 had home internet that was incompatible with the technology used in this study, namely use of a mini router to connect the EchoDot device to the participants home internet service.

Supplementary section S4: Summaries of responses to free-text questionnaire items

Participation questionnaire

Other reason participant took part

Two participants provided a response. One was interested in the application of new technologies to self-reported data, and the other wanted to see what the experience was like for a participant of a project using a novel technology for remote research.

Other comments about your experience supplying dietary information via Alexa / Other things that happened when using Alexa

32 participants provided additional comments. The comments were focused around three broad themes: 1) using the Alexa skill, 2) articulating or remembering/being able to provide information, and 3) and comments relating the study protocol.

Comments relating to using the Alexa skill (from 30 participants): 21 participants commented on the difficulty they had adding items using Alexa, such as difficulty providing Alexa with meals that had several components or non-English meals, and difficulties with inadvertent shutdown of the Alexa skill (e.g., due to a gap in speech). Of these 21 participants, 4 said this meant they simplified what they said so that Alexa could understand, and two said this meant they sometimes did not record whole events. Connection or speed issues were commented on by 12 participants and a further two mentioned times when the skill stopped working. Two participants commented that they would sometimes accidently add their information to the Alexa shopping list skill rather than the study custom skill. Two participants said they preferred writing the information compared with using Alexa while one said they preferred using Alexa. One participant noted the 'now' functionality to record the time did not work for them. 10 participants commented on the sentence structure required by Alexa being difficult, including not allowing pauses in speech, having to say 'add item' which some found inconvenient, and the general learning curve for using the skill. One participant commented that the effort needed meant they reduced the amount they snacked to avoid having to add an entry. Three participants noted they had difficulties using the skill generally, such as with inadvertent interruptions or skill shutdowns. One participant commented that they felt more connected using the web form compared with using the Alexa skill.

Comments relating to articulating or remembering/being able to provide information (from 11 participants): Nine participants commented that it could be difficult to articulate information to Alexa, for example, quantities and ingredients, with one participant who forgot to record quantities, another unsure on the amount of detail that was required, and another who was unsure whether different items in a meal should be in the same event. Three participants commented that they did not include water intake in their diaries, with one of these also noting they also did not add herbal teas as it does not have [many] calories. One participant noted that they did not add diary information on one day due to illness, and another noted that they often did not record their events soon after they occurred.

Comments relating to the study protocol (from 9 participants): Five participants said that it was difficult when they ate or drank away from home, because their Alexa device was at home. Two participants made comments regarding the web form, one that Alexa would have been easier if they did not also need to use the web form, and the other that they did not fill in the web form which they suggested was because Alexa is easier. One participant commented that they hoped they would feel better about providing their food intake because they did not have to type it out, but that they did have to do that using the web form in this study. One participant noted that they did not know if Alexa had recorded what they told them (our study setup did not allow the participants to see the information they provided).

Other reasons participant is not happy to use a voice-controlled system (e.g. Alexa) at home for research in the future?
Five participants provided a response. Of which, four participants commented on the issues they encountered such as having to repeat information and having disruptions when using it. The remaining participant commented that using the wearable would be more convenient compared with using a home-based device.

Other reasons participant is not happy to use a voice-controlled system (e.g. Alexa) on a wearable device such as a smart watch, for research
One participant provided an additional reason, stating that it was because they needed to repeat information.

<u>Non-participation questionnaire</u>
Other reasons given for not taking part, provided in non-participation questionnaire
Of the 21 respondents who provided an additional reason, 15 of these said they did not receive or see the email inviting them to participate in the study. Other reasons given were not meeting the eligibility criteria, being busy or distracted with other things in their lives at the time the study took place, considering participating after recruitment had ended, 'lazyness', and the belief that Alexa only understands English foods.

Other reasons respondent is not happy to use a voice-controlled system (e.g. Alexa) at home for research in the future?
Of the 3 respondents who provided an additional reason, one cited privacy concerns and one talked about the effort required. The other talked about only want to pay for a device if it works well but as devices would be provided this individual has misunderstood.

Supplementary section S5: Further study strengths and limitations

<u>Further strengths</u>
We originally planned a study based at the University, and successfully adapted the study protocol to be 'contactless' so that it could be carried out during the pandemic.

<u>Further limitations</u>
While we provided an information sheet with the Alexa device to help participants to get started with Alexa, more comprehensive training may be useful, for example, providing a training video or asking them to try a test submission in the presence of a researcher who can advise them.

While asking participants to provide information via both Alexa and a web form was valuable, interactions with one of these approaches may have impacted their interaction and perceived feelings

towards the other. Specifically, we asked participants to enter the information using Alexa first and then the web form, so the web form entry might be affected by their interaction with Alexa. For example, if the participant felt they could not enter much detail using Alexa, then they would likely then enter the same level of detail using the web form, but if this was reversed then we might be able to see how the information is reduced/changed due to the voice interface. Likewise, the participants experience using Alexa may have been impacted by having to also complete a web form.

We collected food and drink information via Alexa and the web form with no explicit link between them, asking the participants to enter the intake information via the web form after submitting this to Alexa. Contrary to our expectations, participants often did not enter the web entry directly after the Alexa entry and sometimes submitted them at completely different times. This meant it was difficult to identify the entries of these two approaches that corresponded to the same intake event. Many of the entries were excluded from our comparison of the submitted entries because we could not identify the counterpart entry entered with the other method.

SUPPLEMENTARY FIGURES

Supplementary figure S1: Participant flow diagram

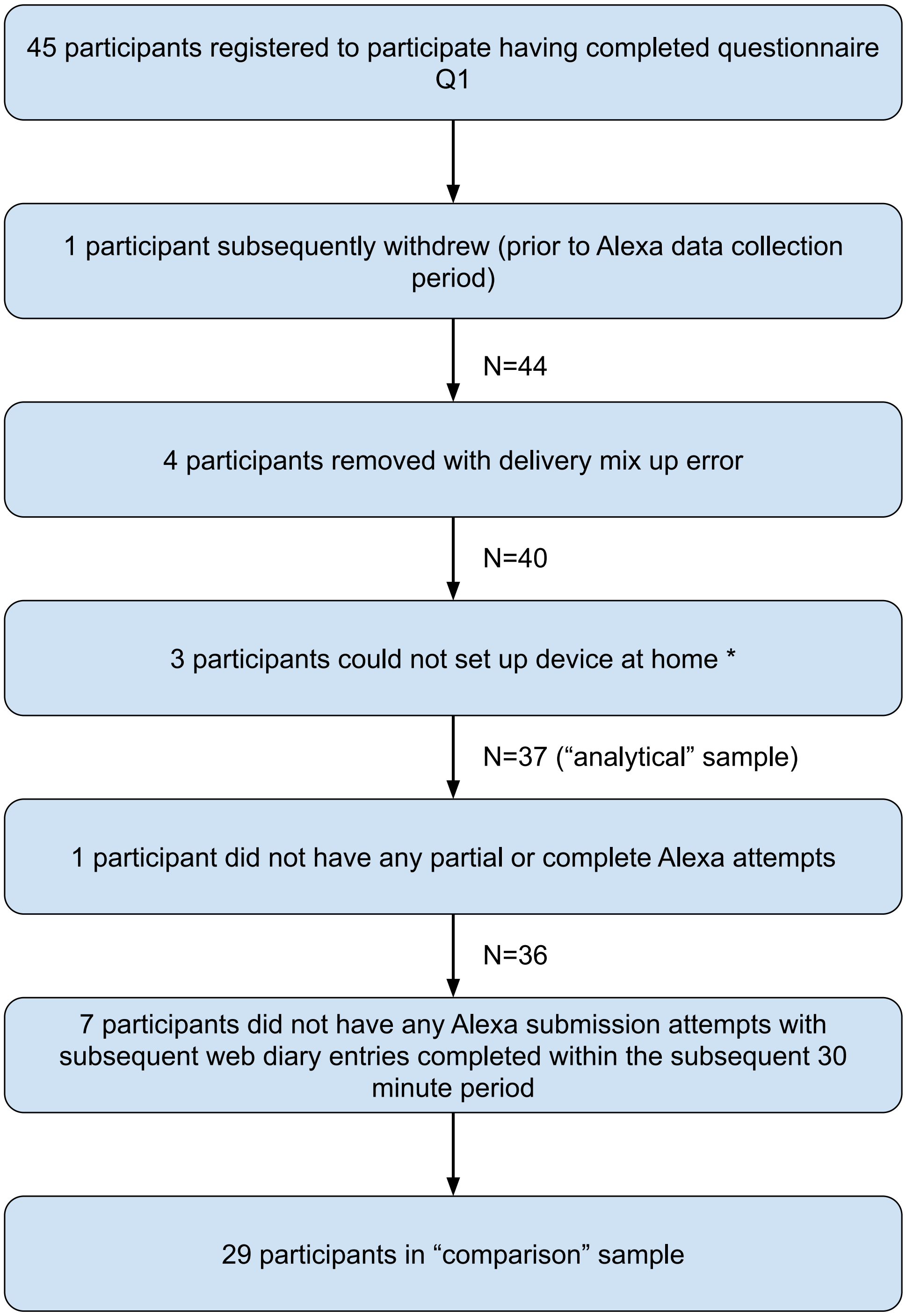

* This was due to the router-based set up of this study rather than due to the Alexa technology, see Supplementary section S3 for further details.

Supplementary figure S2: Infrastructure of custom Alexa skill

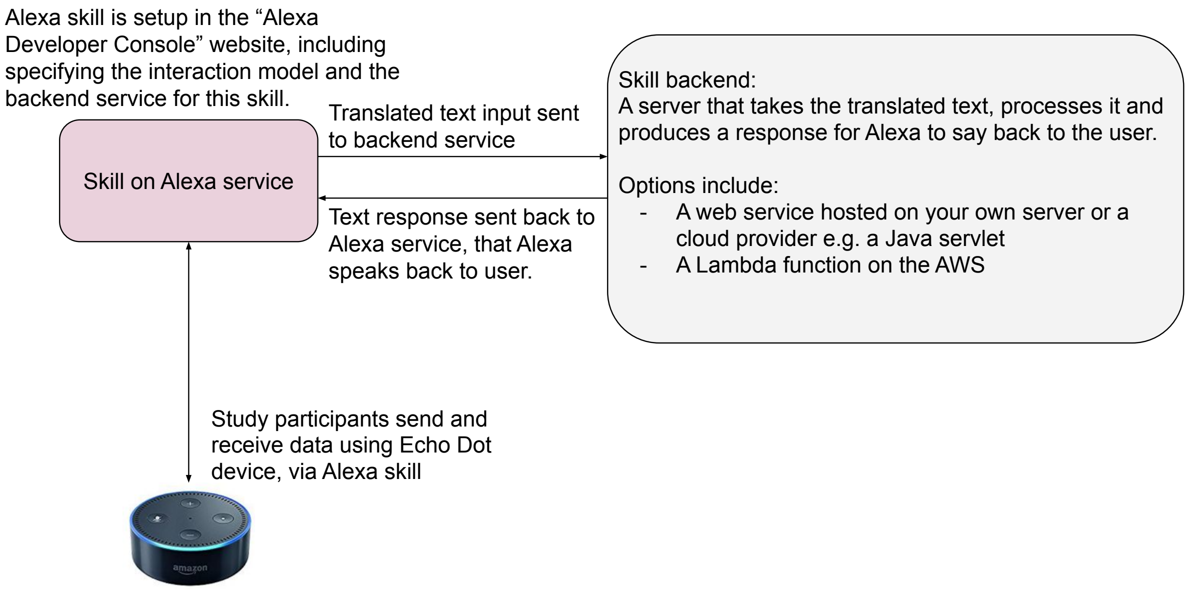

Supplementary figure S3: System architecture

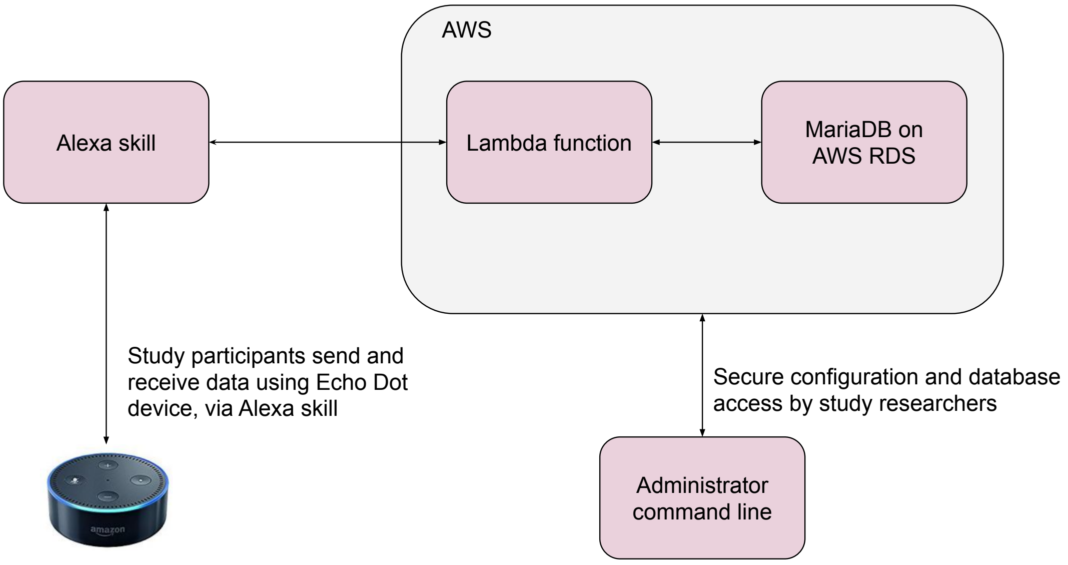

The Amazon Echo Dot device listens for the keyword ‘Alexa’ and then audio following this is sent to the Amazon web service. This web service parses the audio and uses our Alexa ‘skill’ to convert to text information. This textual data is then sent to our web service (hosted as an AWS lambda function), which stores the information in a MariaDB database on the AWS RDS.

Supplementary figure S4: User journey of food diary skill

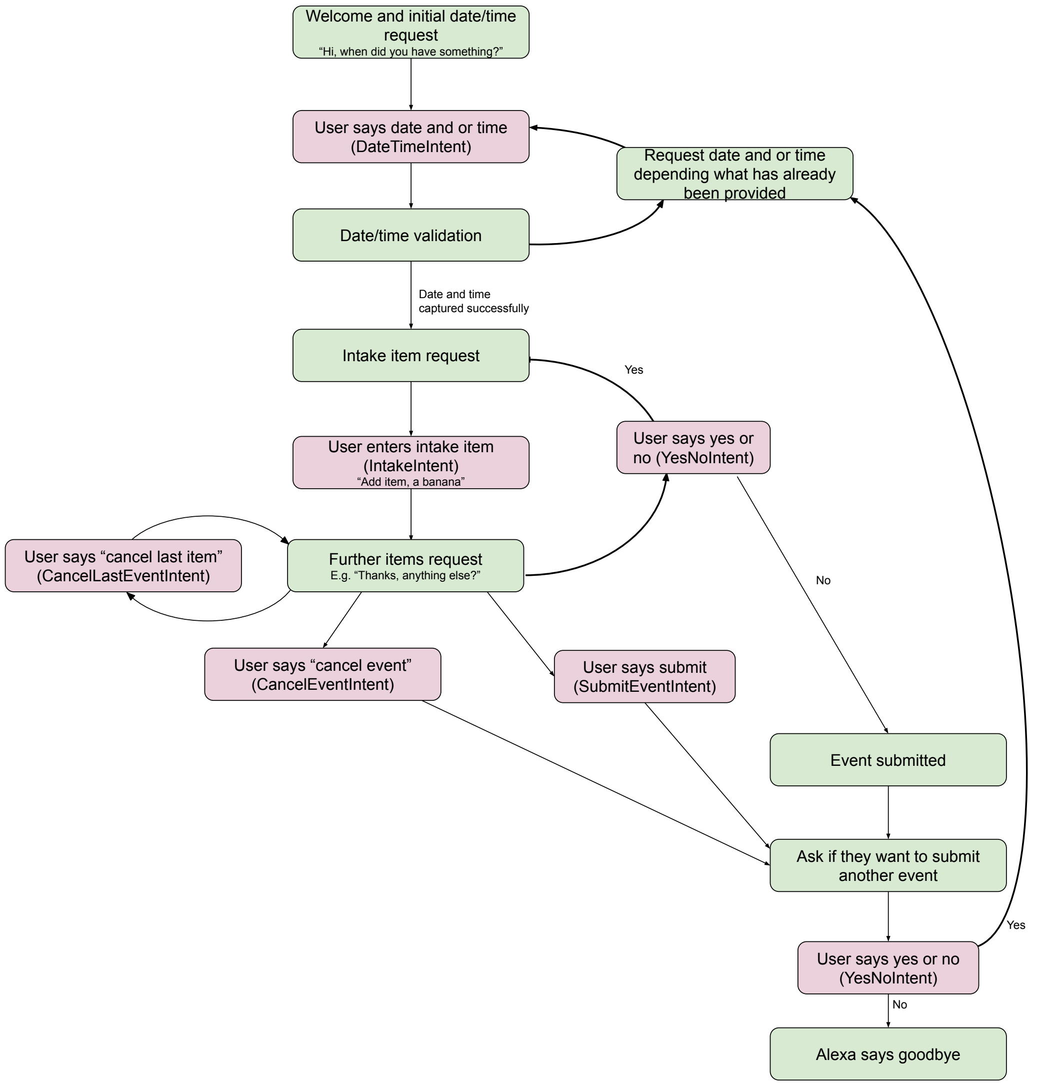

Supplementary figure S5: Food diary data processing approach

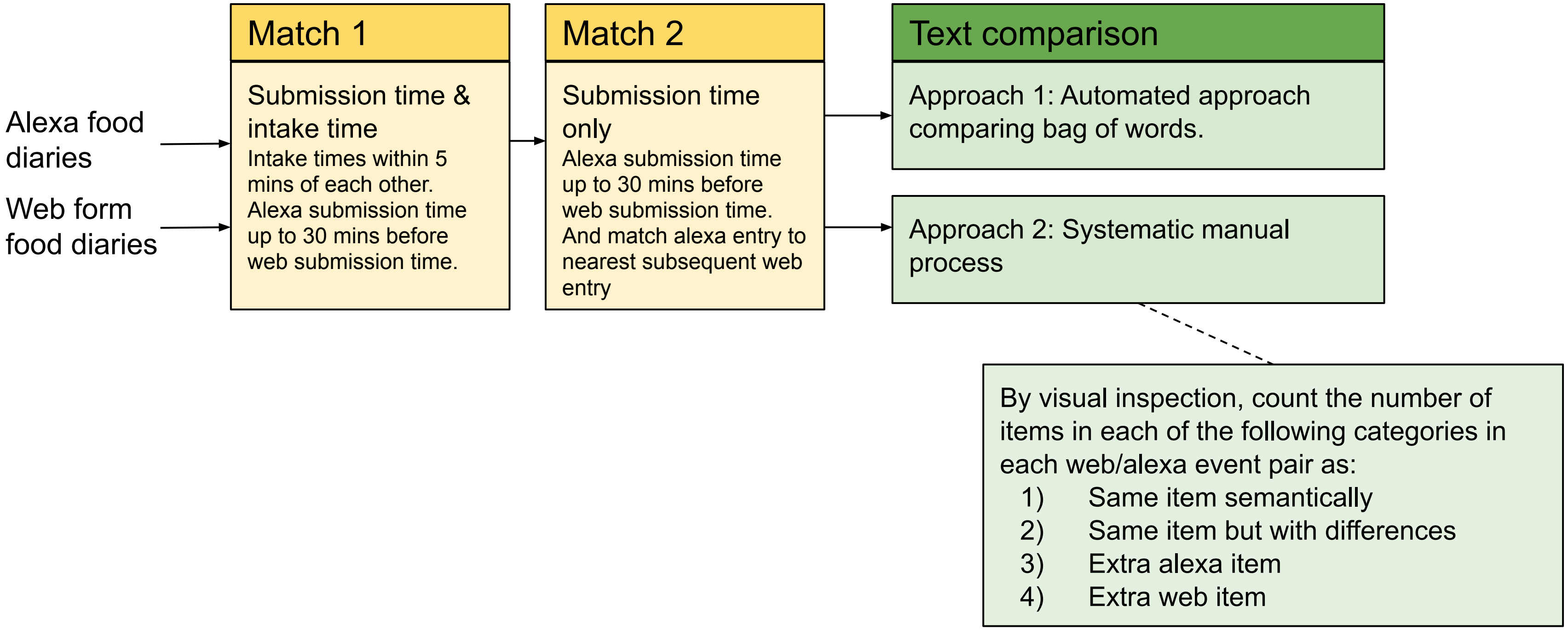

Supplementary figure S6: Summaries of the number of diary entries entered via the web form and Alexa

a) Number of web form entries versus Alexa entries

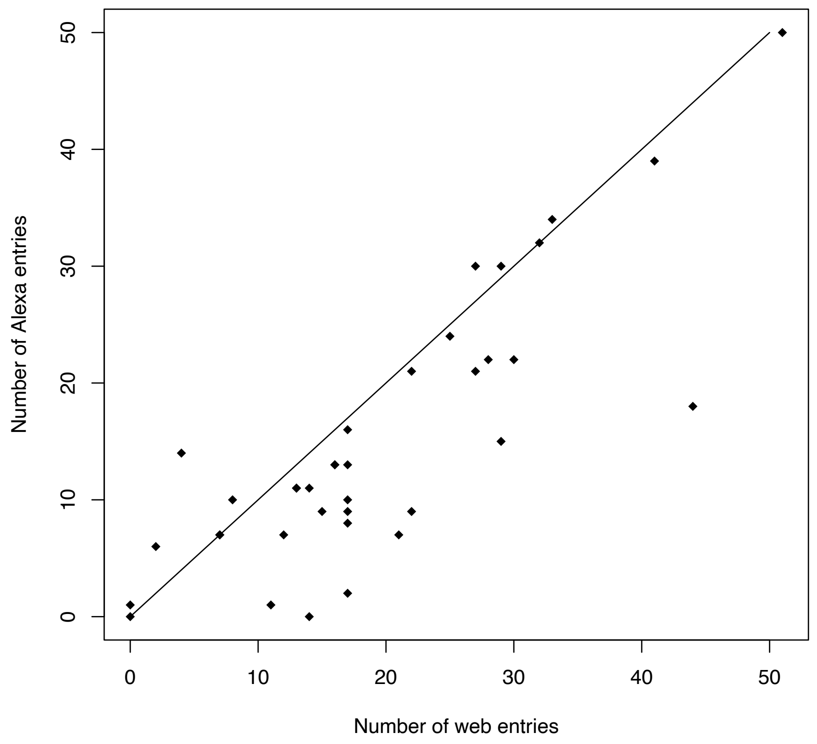

b) Number of partial Alexa entries

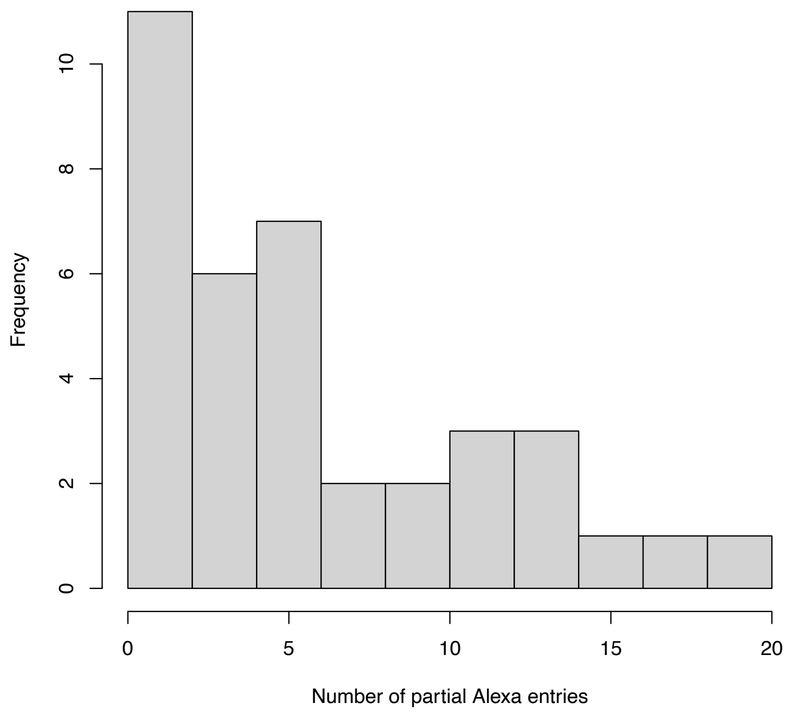

These summaries are of the main analytical sample, N = 37.

Supplementary figure S7: Plot comparing intake timestamps recorded using Alexa versus the web form

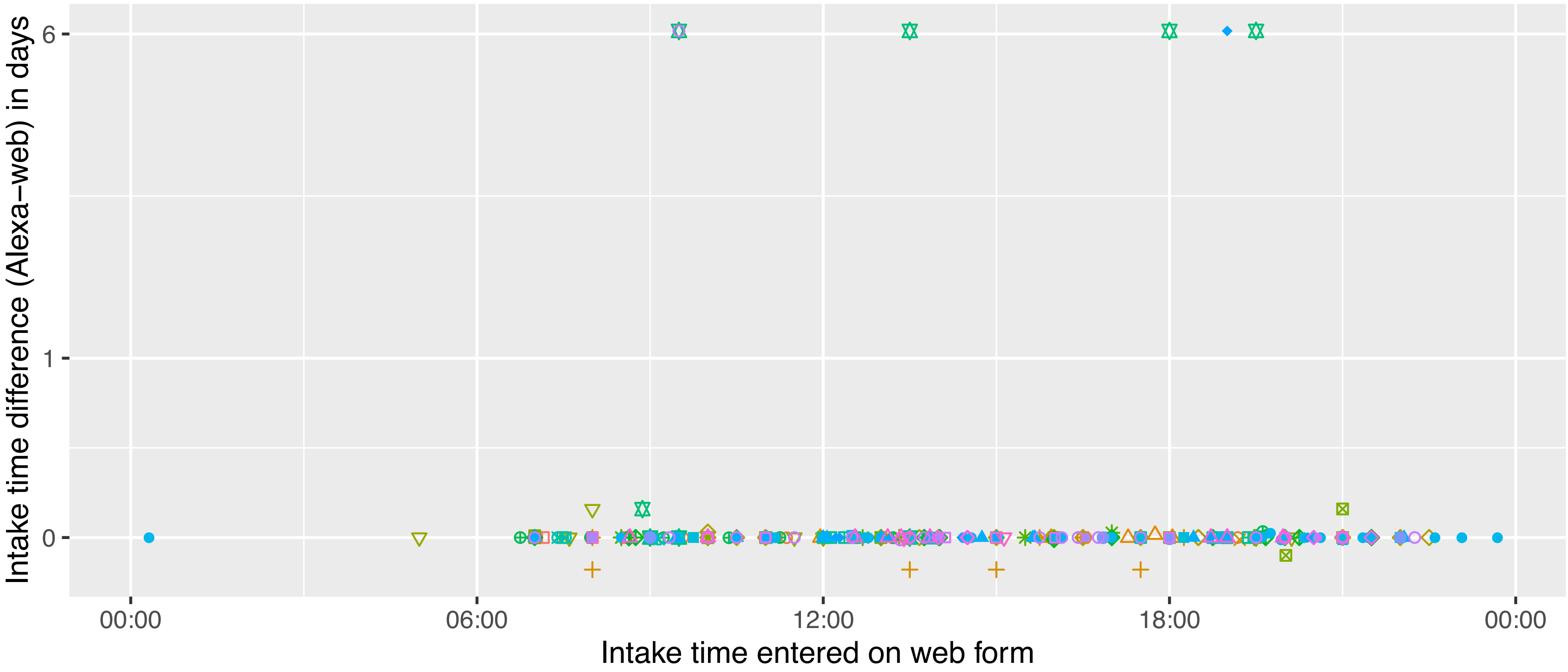

Each colour/symbol combination denotes a particular participant. Each point refers to an intake event for a participant, and shows the intake event time entered using the web form (x-axis) and the difference between the intake event time entered using Alexa versus the web form (y-axis)

Supplementary figure S8: Comparison of systematic manual approach assessments of LACM versus independent assessors

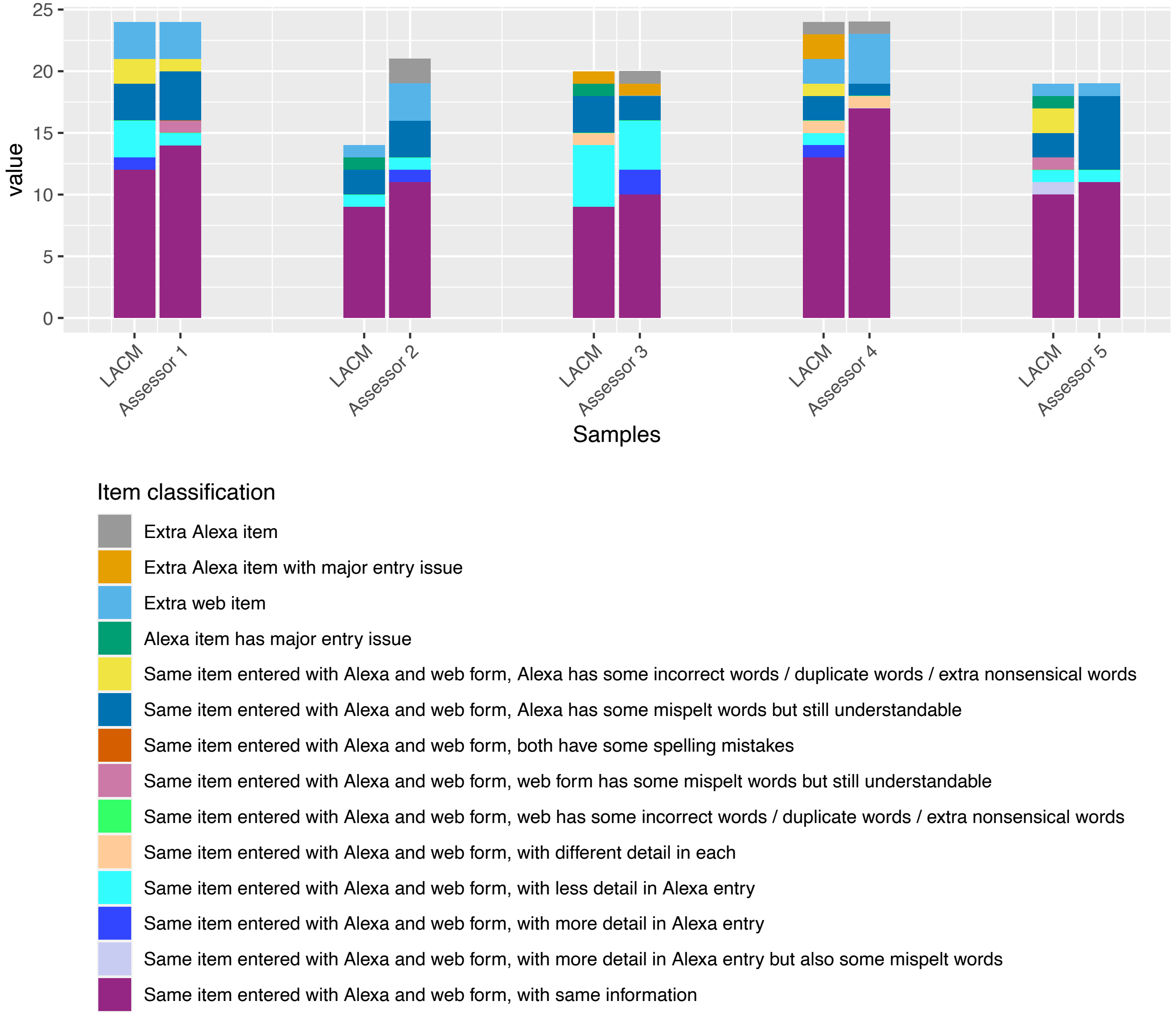

Assessor bars contain the summary of assessments for the set of examples randomly assigned to that assessor. Next to each are the assignments by LACM, for that same set of examples.

SUPPLEMENTARY TABLES

Supplementary table S1: Skill intents and example utterances

**Intent**	**Description**	**Example utterances**
Intake event	Add food or drink item to intake event.	"Add item, a cheese and salad sandwich on brown bread"<br>"Yes add item, a cup of tea"
Date and time	Submit date and time of intake event.	"An hour ago"<br>"Ten thirty"<br>"Yesterday at 4pm"
Cancel event	Cancel the current intake event and start from the beginning.	"Cancel event"<br>"Start over"
Cancel last item	Remove the last intake item from the current intake event.	"Remove last item"<br>"Delete last item"
Yes No	Reply to a yes/no question during the conversational logic. For example, when Alexa asks the participant if they have other items or events to submit (see Figure 1 in main paper).	"Yes"<br>"No"
Submit event	Submit this intake event.	"Submit event"<br>"Submit"

Supplementary table S2: Examples of manual text comparison

Web entry	Alexa entry	Result
**Examples comparing individual items**		
1 x apple	One apple	This is the same item because the meaning is equivalent. Add 1 to *same_item* column.
A bowl of porridge	Some porridge	This is the same item but with extra info in the web entry. Add 1 to *same_item_alexa_less_detail* column.
20g chocolate	20	Here there is no indication that it is referring to a different item, but it is not recognizable as this item. Add 1 to item_with_major_alexa_entry_issue column.
A small fruit bowl	A small football	The alexa item is not recognizable as fruit. Add 1 to item_with_major_alexa_entry_issue column.
Tomato pasta topped with cheese	Tomato pasta topped with cheddar	This is the same item but with different detail ('cheese' vs 'cheddar'). Add 1 to *same_item_alexa_different_detail* column.
A handful of cashew nuts	15 cashew nuts	This is the same item but with different detail ('handful' vs a specific quantity). Add 1 to *same_item_alexa_different_detail* column.
Cup of tea	Cup of tea with milk	This is the same item but with extra info in the alexa entry (i.e. including that the tea was with milk). Add 1 to *same_item_alexa_more_detail* column.
A coffee with milk	A cough with milk	'Coffee' is incorrectly recorded as 'cough' by Alexa. Add 1 to *same_item_alexa_entry_issue* column.
2 x bagels with peanut butter	To bagels with peanut butter	The quantity is recorded by Alexa as 'to' rather than 'two'. Add 1 to *same_item_alexa_entry_issue* column.
**Example comparing whole intake event**		
20g chocolate<br>A coffee with milk<br>A bagel with peanut butter	A cough with milk<br>20	Step 1: Note that there are 3 web items but only 2 Alexa items. Add this to the *total_web_items* and *total_alexa* items columns of the spreadsheet.<br><br>Step 2: Match the items that most likely correspond to each other:<br>"A coffee with milk" -> "A cough with milk"<br>Add 1 to the same_item_alexa_mispelt column.<br><br>"20g chocolate" -> "20"<br>While the Alexa entry is sparse, it has some similarity with the chocolate web item so this is the most likely corresponding item. Something has gone wrong entering this information with Alexa, so add 1 to the *item_with_major_alexa_entry_issue* column.<br><br>3. Identify the items that cannot be matched. In this case, it is the "A bagel with peanut butter" web entry. Add 1 to the *extra_web_item* column.

Supplementary table S3: Post-participation questionnaire summary

Question	Total N	N (%)
Reasons did take part – a) Interested in nutritional research	35	18 (51%)
– b) Wanted to support research.	35	27 (77%)
– c) Wanted to support others in the same research unit	35	12 (34%)
– d) Felt should be a research participant when asked, as they benefit from other participants	35	11 (31%)
– e) Wanted to try a voice-controlled system	35	7 (20%)
– f) Wanted to try Alexa	35	12 (34%)
– g) Wanted to see how well Alexa could interact with them.	35	12 (34%)
– h) Wanted to see how well Alexa could record their dietary information.	35	17 (49%)
– i) Other reason [a]	35	2 (6%)
Participant was able to accurately tell Alexa what they ate and drank: – Completely agree	35	3 (9%)
– Somewhat agree		16 (46%)
– Neither agree not disagree		2 (6%)
– Somewhat disagree		12 (34%)
– Completely disagree		2 (6%)
Participant was able to estimate accurate quantities describing how much they ate – Completely agree	34	3 (9%)
– Somewhat agree		11 (32%)
– Neither agree not disagree		10 (29%)
– Somewhat disagree		9 (26%)
– Completely disagree		1 (3%)
Participant chose not to record particular snacks/meals (e.g. because it was unhealthy) – Completely agree	35	1 (3%)
– Somewhat agree		5 (14%)
– Neither agree not disagree		0 (0%)
– Somewhat disagree		7 (20%)
– Completely disagree		22 (63%)
Participant sometimes chose to be selective with the truth – Completely agree	35	3 (9%)
– Somewhat agree		5 (14%)
– Neither agree not disagree		3 (9%)
– Somewhat disagree		6 (17%)
– Completely disagree		18 (51%)
Participant felt they remembered to submit food and drink information – Completely agree	35	9 (26%)
– Somewhat agree		19 (54%)
– Neither agree not disagree		1 (3%)
– Somewhat disagree		6 (17%)
– Completely disagree		0 (0%)
Other comments about your experience supplying dietary information via Alexa *	35	29 (83%)
Alexa interjected when I hadn't finished telling her when I ate or drank – Never	35	4 (11%)
– Rarely		13 (37%)
– Occasionally		11 (31%)
– Often		6 (17%)
– Always		1 (3%)
Alexa interjected when I hadn't finished telling her what I ate or drank – Never	35	2 (6%)
– Rarely		3 (9%)
– Occasionally		12 (34%)
– Often		17 (49%)
– Always		1 (3%)

Other things that happened when using Alexa [a]	35	30 (86%)
How convenient or inconvenient did you find providing information using Alexa? – Very inconvenient	35	4 (11%)
– Somewhat inconvenient		13 (37%)
– OK		10 (29%)
– Somewhat convenient		7 (20%)
– Very convenient		1 (3%)
How enjoyable or unenjoyable did you find providing information using Alexa? – Very unenjoyable	35	3 (9%)
– Somewhat unenjoyable		11 (31%)
– OK		15 (43%)
– Somewhat enjoyable		6 (17%)
– Very enjoyable		0 (0%)
How efficient or inefficient did you find providing information using Alexa? – Very inefficient	35	5 (14%)
– Somewhat inefficient		15 (43%)
– OK		6 (17%)
– Somewhat efficient		8 (23%)
– Very efficient		1 (3%)
How easy or hard did you find providing information using Alexa? – Could not use at all	35	0 (0%)
– Very hard		3 (9%)
– Somewhat hard		18 (51%)
– OK		7 (10%)
– Somewhat easy		7 (20%)
– Very easy		0 (0%)
Happy to use a voice-controlled system (e.g. Alexa) at home for research in the future: – Yes	35	26 (74%)
– No		2 (6%)
– Not sure		7 (20%)
Not happy reasons: – Data privacy concerns around researchers collecting information about me	35	0 (0%)
– Data privacy concerns around Amazon collecting information about me.	35	2 (6%)
– Concerns that Alexa will inadvertently listen to other conversations, which may then be accessed by researchers	35	1 (3%)
– Concerns that the system will inadvertently listen to other conversations, which may then be used by the company that owns it (e.g. Amazon)	35	3 (9%)
– Other reason [a]	35	5 (14%)
Happy to use a voice-controlled system (e.g. Alexa) on a wearable device such as a smart watch, for research – Yes	35	28 (80%)
– No		1 (3%)
– Not sure		6 (17%)
Not happy reasons: – Data privacy concerns around researchers collecting information about me	35	1 (3%)
– Data privacy concerns around Amazon collecting information about me.	35	1 (3%)
– Concerns that Alexa will inadvertently listen to other conversations, which may then be accessed by researchers	35	1 (3%)
– Concerns that the system will inadvertently listen to other conversations, which may then be used by the company that owns it (e.g. Amazon)	35	3 (9%)
– I wouldn't like the idea of wearing a device all the time because of how it looks and / or feels.	35	2 (6%)
– Other reason [a]	35	1 (3%)
**Other approaches to collect dietary data**		
Participant has used another approach to record food and drink diary	35	25 (71%)
Participant has used a traditional diary	35	13 (37%)

Alexa Convenience compared with traditional diary – Much less convenient	13	3 (23%)
– A little less convenient		3 (23%)
– About the same		1 (8%)
– A little more convenient		5 (38%)
– Much more convenient		1 (8%)
Alexa how enjoyable compared with traditional diary – Much less enjoyable	9	2 (22%)
– A little less enjoyable		2 (22%)
– About the same		1 (11%)
– A little more enjoyable		3 (33%)
– Much more enjoyable		1 (11%)
Alexa efficiency compared with traditional diary – Much less efficient	9	4 (44%)
– A little less efficient		3 (33%)
– About the same		0 (0%)
– A little more efficient		1 (11%)
– Much more efficient		1 (11%)
Participant has used my fitness pal app on phone or tablet	35	12 (34%)
Alexa convenience compared with myfitnesspal app on phone or tablet: – Much less convenient	12	3 (25%)
– A little less convenient		0 (0%)
– About the same		3 (25%)
– A little more convenient		6 (50%)
– Much more convenient		0 (0%)
Alexa how enjoyable compared with myfitnesspal app on phone or tablet – Much less enjoyable	11	2 (18%)
– A little less enjoyable		2 (18%)
– About the same		2 (18%)
– A little more enjoyable		5 (45%)
– Much more enjoyable		0 (0%)
Alexa efficiency compared with myfitnesspal app on phone or tablet – Much less efficient	11	3 (27%)
– A little less efficient		4 (36%)
– About the same		1 (9%)
– A little more efficient		0 (0%)
– Much more efficient		3 (27%)
Participant has used another method, or did not specify the method	35	5 (14%)

[a] Participants who stated ‘other’ were able to then complete a free-text response, and these are summarized in Supplementary Section S4.

Supplementary table S4: Non-participation questionnaire responses

Participant characteristic	N (%)
Age band – <20	13 (19%)
– 20-29	36 (52%)
– 30-39	11 (16%)
– 40-49	5 (7%)
– 50-59	2 (3%)
– Prefer not to answer	2 (3%)
Sex – N female	51 (74%)
Reasons did not take part<br>– Not available during the study session times	30 (43%)
– Data privacy concerns around Amazon collecting information on my diet	9 (13%)
– Data privacy concerns around researchers collecting information on my diet	3 (4%)
– Concerns that Alexa will inadvertently listen to other conversations	9 (13%)
– I don't eat or drink during my working hours	3 (4%)
– Picking up and returning the device was inconvenient	5 (7%)
– Other reason [a]	21 (30%)
Has a voice-controlled device – No	35 (51%)
– Yes but it's used by others, not me	10 (14%)
– Yes and I use it	24 (35%)
Happy to use a voice-controlled system (e.g. Alexa) at home for research in the future - Yes	42 (61%)
- No	13 (19%)
- Not sure	14 (20%)
Not happy reasons:<br>– Data privacy concerns around researchers collecting information about me	10 (14%)
– Data privacy concerns around Amazon collecting information about me.	21 (30%)
– Concerns that Alexa will inadvertently listen to other conversations, which may then be accessed by researchers	16 (23%)
– Concerns that the system will inadvertently listen to other conversations, which may then be used by the company that owns it (e.g. Amazon)	20 (29%)
– Other reason [a]	3 (4%)
Happy to use a voice-controlled system (e.g. Alexa) on a wearable device such as a smart watch, for research – Yes	39 (57%)
– No	18 (26%)
– Not sure	12 (17%)
Not happy reasons:<br>– Data privacy concerns around researchers collecting information about me	8 (12%)
– Data privacy concerns around Amazon collecting information about me.	19 (28%)
– Concerns that Alexa will inadvertently listen to other conversations, which may then be accessed by researchers	15 (22%)
– Concerns that the system will inadvertently listen to other conversations, which may then be used by the company that owns it (e.g. Amazon)	19 (28%)
– I wouldn't like the idea of wearing a device all the time because of how it looks and / or feels.	16 (23%)
– Other reason	0 (0%)

N = 69.
[a] Participants who stated 'other' were able to then complete a free-text response, and these are summarized in Supplementary Section S4.
